# An efficient method for the construction of polysubstituted 4-pyridones via self-condensation of β-keto amides mediated by P_2_O_5_ and catalyzed by zinc bromide

**DOI:** 10.3762/bjoc.9.304

**Published:** 2013-11-28

**Authors:** Liquan Tan, Peng Zhou, Cui Chen, Weibing Liu

**Affiliations:** 1 School of Chemistry and Life Science, Guangdong University of Petrochemical Technology, 2 Guangdu Road, Maoming 525000, China

**Keywords:** β-keto amide, phosphorus pentoxide, 2-pyridones, 4-pyridones, self-condensation

## Abstract

A self-condensation cyclization reaction mediated by phosphorus pentoxide (P_2_O_5_) and catalyzed by zinc bromide (ZnBr_2_) is presented for the synthesis of polysubstituted 4-pyridones and 2-pyridones from β-keto amides. A variety of β-keto amides are used in this approach, and a wide range of functionalized 4-pyridones and 2-pyridones were obtained in good to excellent yields. When employing the *N*-aryl β-keto amides as the substrates in this protocol, 4-pyridones are resulted, however, when using *N*-aliphatic-substituted β-keto amides as the partners of *N*-aryl β-keto amides under the same conditions, 2-pyridones are afforded.

## Introduction

**Scheme 1:**
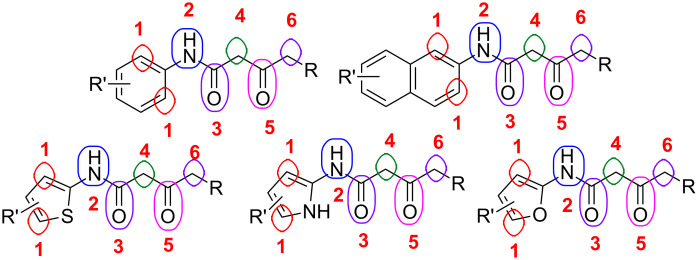
The six different reactive positions of β-keto amides.

β-Keto amide and their derivatives are desired classes of intermediates for the synthesis of nitrogen- and oxygen-containing heterocyclic compounds since they possess six reactive sites in the same molecule (Scheme 1) [1–7]. A lot of reports can be found in the literature concerning the construction of different heterocyclic compounds from β-keto amides by modification of the six different reactive positions [8–13]. Our interest in the fields of cleavage or construction of C–C and C–hetero bonds using β-keto amides and their derivatives as the substrates prompted us to exploit the reactivity of the six different reactive positions of β-keto amides [14–18].

**Scheme 2:**
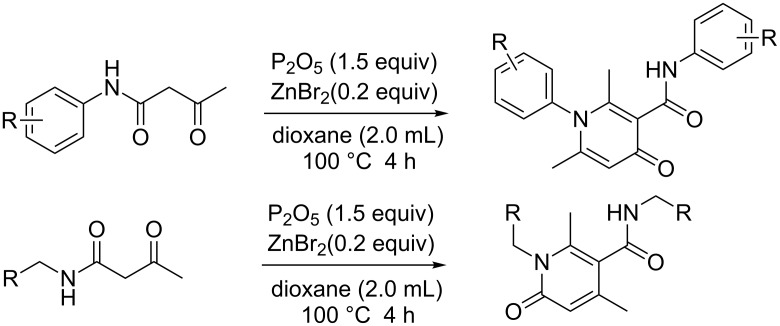
Synthesis of polysubstituted 4-pyridones from β-keto amides.

Two groups reported the self-condensation of *N*-aryl β-keto amides. The group of Zhang used Na_2_S_2_O_8_ as the reagent to induce this condensation [19] and the group of Ovenden used tosic acid to catalyze this transformation [20]. In this article, we report an improved efficient method for the construction of polysubstituted 4-pyridones and 2-pyridones via phosphorus pentoxide-mediated self-condensation of β-ketobutylanilides catalyzed by zinc bromide (Scheme 2). It is well known that 4-pyridones are one of the most important classes of heterocyclic compounds as they are a key structural attribute of many bioactive natural products [21–27]. Besides, many of the 4-pyridones have shown interesting biological activities, such as antibacterial [24,28], antitumor [29], antiviral [30], and several other potentially useful activities [27,31].

## Results and Discussion

In our initial study, 3-oxo-*N*-phenylbutanamide (**1a**) was selected as a model substrate to optimize the reaction conditions (Table 1). The preliminary results showed that this self-condensation cyclization reaction did not occur in the absence of ZnBr_2_ (Table 1, entry 1) or P_2_O_5_ (Table 1, entry 3) at room temperature. Notable efficacy was achieved when increasing the reaction temperature to 100 °C for 4 h and afforded the desired product 1,4-dihydro-2,6-dimethyl-4-oxo-*N*,1-diphenylpyridine-3-carboxamide (**2a**) in 94% GC yield (Table 1, entries 2, 4–6). Results of the screening study of the amount of ZnBr_2_ and P_2_O_5_ (Table 1, entries 6–8 and 12) indicated that 0.2 equiv of ZnBr_2_ and 1.5 equiv of P_2_O_5_ was sufficient for the completion of this transformation. In order to improve the efficiency of the reaction, we tested several zinc salts for this reaction (Table 1, entries 7, 9–11). The use of ZnBr_2_ and ZnCl_2_ greatly facilitated the reaction, and both gave **2a** in excellent GC yield (Table 1, entries 7 and 9). Among the various solvents examined (Table 1, entries 7, 13–17), dioxane proved to be the most suitable solvent for this transformation (Table 1, entry 7). After screening, the optimal reaction conditions were obtained; these are, the mixture of 3-oxo-*N*-phenylbutanamide **1a** with 0.2 equiv of ZnBr_2_ and 1.5 equiv of P_2_O_5_ reacted in dioxane (2.0 mL) at 100 °C for 4 h (Table 1, entry 7).

**Table 1:** Optimization of reaction conditions^a^.

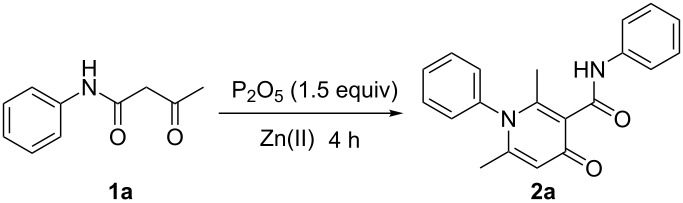				

Entry	Solvent	Zn(II) (equiv)	Temp. ( °C)	Yield (%)^b^

1^c^	Dioxane	0	rt	0
2^c^	Dioxane	ZnBr_2_ (1.0)	rt	13
3^c,d^	Dioxane	ZnBr_2_ (1.0)	rt	0
4^e^	Dioxane	ZnBr_2_ (1.0)	100	72
5	Dioxane	ZnBr_2_ (1.0)	100	94
6^c^	Dioxane	ZnBr_2_ (1.0)	100	94
7	Dioxane	ZnBr_2_ (0.2)	100	94
8	Dioxane	ZnBr_2_ (0.5)	100	94
9	Dioxane	ZnCl_2_ (0.2)	100	92
10	Dioxane	Zn(OAc)_2_ (0.2)	100	45
11	Dioxane	ZnO (0.2)	100	0
12^f^	Dioxane	ZnBr_2_ (0.2)	100	84
13	Cyclohexane	ZnBr_2_ (0.2)	reflux	56
14	DCE	ZnBr_2_ (0.2)	reflux	81
15	DMF	ZnBr_2_ (0.2)	100	32
16	AcOH	ZnBr_2_ (0.2)	100	14
17	MeOH	ZnBr_2_ (0.2)	reflux	30

^a^All reactions were carried out with **1a** in 0.25 mmol scale and 2 mL solvent; ^b^GC yield; ^c^reaction time: 6 h; ^d^without P_2_O_5_; ^e^reaction time: 2 h; ^f^P_2_O_5_: 1.0 equiv.

**Scheme 3:**
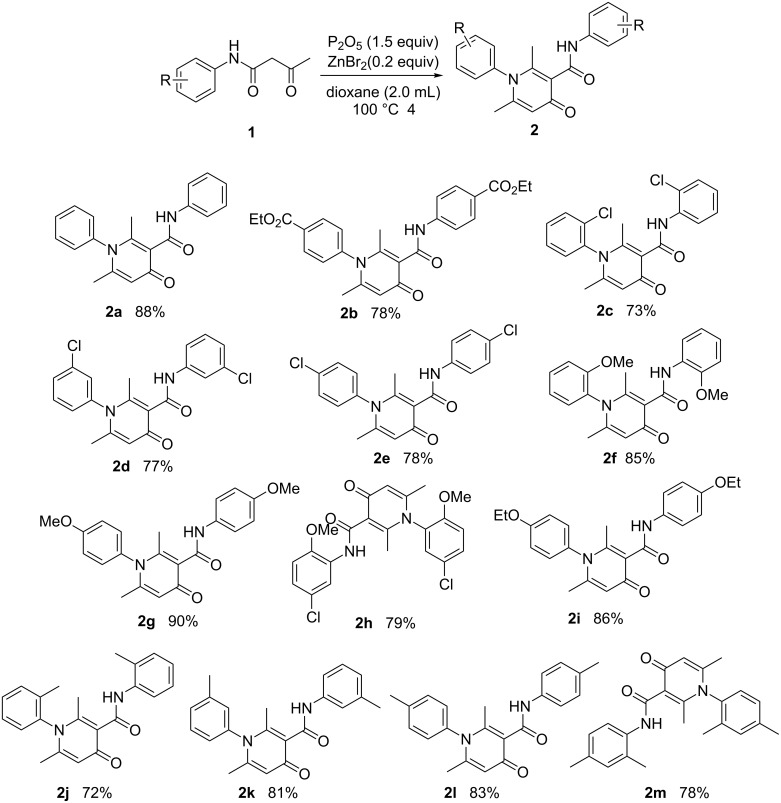
The scope of the substrates. (Note: All the listed yields are isolated yields.)

To explore the substrate scope and limitations of this self-condensation cyclization reaction, a range of N-aryl β-keto amides were then examined under the optimal reaction conditions. The results are shown in Scheme 3.

**Scheme 4:**
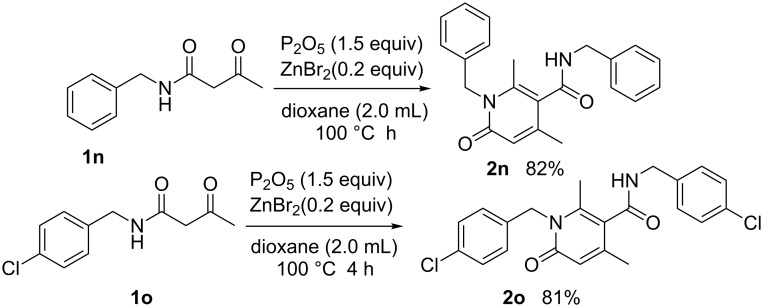
Synthesis of polysubstituted 4-pyridones from *N*-aliphatic-substituted β-keto amides.

The results indicated that a variety of substituted N-aryl acetoacetamides **1a**–**1k** could be easily converted into their corresponding 4-pyridone **2a***–***2k**. All the reactions proceeded smoothly and afforded the desired product in good to excellent isolated yields (72–90%) in 4 h. This transformation appears quite tolerant with respect to the positions of the substituents on the aryl group (*para*-, *meta*- and *ortho*-positions). For example, the reactions of *N*-(2-chlorophenyl)-3-oxobutanamide (**1c**), *N*-(3-chlorophenyl)-3-oxobutanamide (**1d**), *N*-(4-chlorophenyl)-3-oxobutanamide (**1e**), as well as 3-oxo-*N*-*o*-tolylbutanamide (**1j**), 3-oxo-*N*-*m*-tolylbutanamide (**1k**) and 3-oxo-*N*-*p*-tolylbutanamide (**1l**) all lead to their corresponding product (**2c, 2d, 2e, 2j, 2k** and **2l**) in good isolated yield. Furthermore, various electron-withdrawing groups (EWG) (Cl and CO_2_Et) and electron-donating groups (EDG) (Me, OMe and OEt) on the benzene ring had no obvious influence on the reaction, including without a substituent or with both an EWG and EDG substituent on the benzene ring (Scheme 3), all could be easily converted into their corresponding product with good isolated yield. Besides, the scope of this protocol was further examined by the *N*-aliphatic-substituted β-keto amides (**1n** and **1o**) (Scheme 4). We found that the reactions did not afford the 4-pyridones, but provided 2-pyridones **2n** and **4o** with 82% and 81% isolated yield, respectively.

**Scheme 5:**
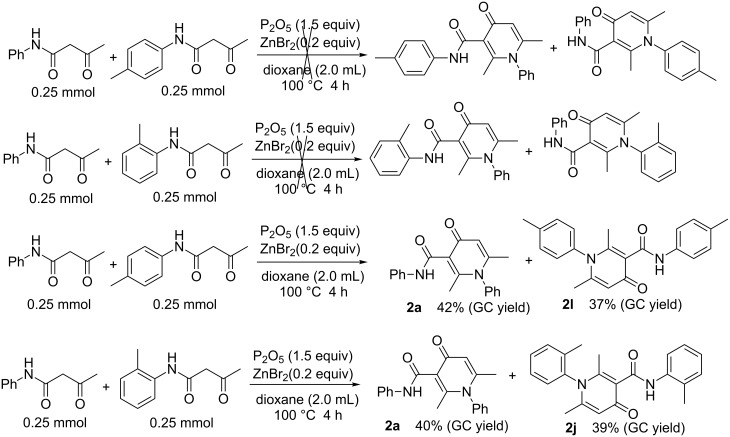
Construct the cross-condensation products.

What is the result when employing different β-keto amides? Is it possible to achieve the cross-condensation products? Therefore, we attempted the reactions under the same conditions (Scheme 5). We were astonished to find that the results only included the self-condensation products (**2a, 2l, 2j**) and achieved not the cross-condensation products.

**Scheme 6:**
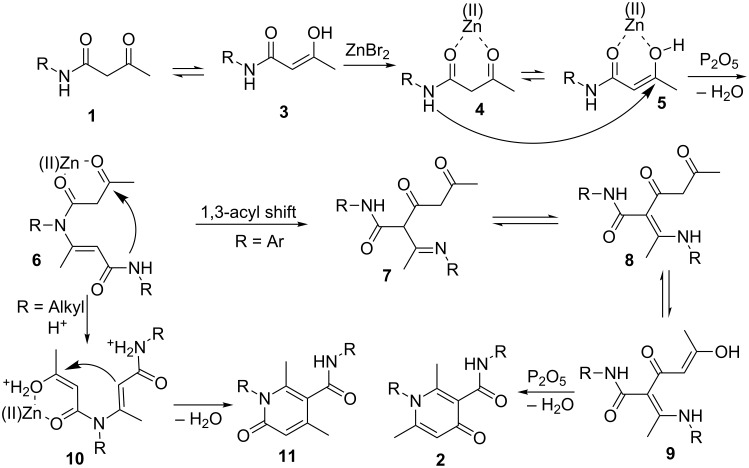
Hypothesized mechanism.

It is well known that Lewis acids can activate β-keto amides [32]. Under mild conditions, phosphorus pentoxide (P_2_O_5_) is often used as the dehydrating agent in organic synthesis for the condensation of amines with carbonyl compounds [33–34]. Based on these evidences, a plausible reaction pathway of this self-condensation cyclization reaction was hypothesized as shown in Scheme 6. The first step of this transformation involved the coordination of β-keto amides **1** with ZnBr_2_ to activate the carbonyl group and to form intermediates **4** and **5**, which was followed by the attack of the lone-pair electrons of the amide nitrogen to the carbonyl carbon in the presence of P_2_O_5_ and intermediate **6** was formed by eliminating one equivalent of H_2_O. Next, when R is aryl, a 1,3-acyl migration occurred from N to C of intermediate **6** [35–39], and affords imine **7** and its equilibrium compound **8**, the subsequent intramolecular nucleophilic cyclization of intermediate **8** provides the final product 4-pyridones [20,38]. Alternatively, when R is aliphatic, the 1,3-acyl migration is not observed – maybe the migration leads to an unstable imine intermediate. Therefore, under the acidic conditions, a cationic species **10** is generated from the intermediate **6** and the subsequent intramolecular dehydration process provides 2-pyridones **11** [19].

## Conclusion

In summary, we have established an improved efficient synthetic protocol for the synthesis of 4-pyridone derivatives by a sequence of intermolecular dehydration of β-keto amides in the presence of phosphorus pentoxide (P_2_O_5_), 1,3-acyl migration and intramolecular dehydration. Comparing to the similar findings, the simple operation with satisfactory yields, relatively cheap additive P_2_O_5_ and zinc catalyst, and the mild reaction conditions are the advantages of this protocol. Further investigations concerning the scope of this reaction, applications, and mechanistic details are currently on-going in our laboratory.

## Supporting Information

File 1 Full experimental details and copies of NMR spectral data.
