# Supplementary material for: An efficient method for the construction of polysubstituted 4-pyridones via self-condensation of β-keto amides mediated by P2O5 and catalyzed by zinc bromide
Source: Beilstein J Org Chem. 2013 Nov 28;9:2681–7. doi: 10.3762/bjoc.9.304 (PMC3869214; doi:10.3762/bjoc.9.304)
Supplement: File 1 — Full experimental details and copies of NMR spectral data. [file Beilstein_J_Org_Chem-09-2681-s001.pdf]

Supporting Information

for

An efficient method for the construction of polysubstituted 4-pyridones via self-condensation of β-keto amides mediated by P_2O_5 and catalyzed by zinc bromide

Liquan Tan*, Peng Zhou, Cui Chen and Weibing Liu*

Address: School of Chemistry and Life Science, Guangdong University of Petrochemical Technology, 2 Guangdu Road, Maoming 525000, China

Email: Liquan Tan - mmtlq522@gmail.com; Weibing Liu - lwb409@gmail.com

*Corresponding author

Full experimental details and copies of NMR spectral data

General methods. All the reactions were carried out at 100 °C for 4 h in a Schlenk tube equipped with magnetic stir bar. Solvents and reagents were purchased from Aldrich Chemicals or J & K Scientific Ltd, and were used as received. Petroleum ether (PE) refers to the fraction boiling in the 60–90 °C range. Thin-layer chromatography was performed using Qingdao-Haiyang 600 mesh silica gel plates (GF254), and samples were made visual with short-wavelength UV light (254 nm). Melting points were measured on a melting point apparatus equipped with a thermometer and were uncorrected. IR spectra were recorded on a Bruker Vector 22 spectrometer as KBr pellets. ^{1}H NMR and ^{13}C NMR spectra were recorded on a 400 MHz spectrometer in solutions of $CDCl_3$ using tetramethylsilane as the internal standard, δ values are given in ppm and coupling constants (*J*) in Hz. GC–MS was obtained using an electron ionization (EI) Agilent 6890N/5973 mass spectrometer.

Typical procedure for the synthesis of 1,4-dihydro-2,6-dimethyl-4-oxo-*N*,1-diphenylpyridine-3-carboxamide (2a). A mixture of 3-oxo-*N*-phenylbutanamide (**1a**) (177 mg, 1.0 mmol), P_2O_5 (213 mg, 1.5 mmol), $ZnBr_2$ (45 mg, 0.2 mmol) and dioxane (2.0 mL) was added successively in Schlenk tube. After stirring for 4 h at 100 °C, the solution was directly subjected to isolation by PTLC (GF_{254}), eluted with a 10:7 petroleum ether/ethyl acetate mixture to afford the desired product (**2a**) as colorless crystals (140 mg, 88%).

Typical procedure for the synthesis of 1,4-dihydro-2,6-dimethyl-4-oxo-*N*,1-dibenzylpyridine-3-carboxamide (2n). A mixture of 3-oxo-N-phenylbutanamide (**1n**) (191 mg, 1.0 mmol), P_2O_5 (213 mg, 1.5 mmol), $ZnBr_2$ (45 mg, 0.2 mmol) and dioxane (2.0 mL) was added successively in Schlenk tube. After stirring for 4 h at 100 °C, the solution was directly subjected to isolation by PTLC (GF_{254}), eluted with a 10:9 petroleum ether / ethyl acetate mixture to afford the desired product (**2n**) as a colorless crystal (142 mg, 82%).

Characterization data for all prepared compounds:

1,4-Dihydro-2,6-dimethyl-4-oxo-*N*,1-diphenylpyridine-3-carboxamide (2a) [1]

Colorless crystal (140 mg, 88%); Mp: 208.2−210 °C (lit. 209−212 °C); ^{1}H NMR ($CDCl_3$, 400 Hz) δ 12.51 (s, 1H), 7.71-7.68 (d, *J* = 8.0 Hz, 2H), 7.59-7.54 (m, 3H), 7.32-7.28 (t, *J* = 7.6 Hz, 2H), 7.21-7.19 (d, *J* = 7.6 Hz, 2H), 7.07-7.04 (t, *J* = 7.2 Hz, 1H), 6.61 (s, 1H), 2.49 (s, 3H), 1.91 (s, 3H); ^{13}C NMR ($CDCl_3$, 100 Hz) δ 171.6, 164.2, 158.2, 147.5, 139.5, 139.0, 130.5, 130.1, 128.8, 127.6, 123.7, 120.6, 118.4, 21.7, 21.0.

Ethyl 4-(1-(4-(ethoxycarbonyl)phenyl)-2,6-dimethyl-4-oxo-1,4-dihydropyridine-3-carboxamido)benzoate (2b) [2]

Colorless crystal (360 mg, 78%); Mp: 180−182 °C (lit. 179−181 °C); ^{1}H NMR ($CDCl_3$, 400 Hz) δ 13.01 (s, 1H), 8.27-8.25 (d, *J* = 8.0 Hz, 2H), 8.01-7.99 (d, *J* = 8.4 Hz, 2H), 7.81-7.79 (d, *J* = 8.0 Hz, 2H), 7.36-7.34 (d, *J* = 8.4 Hz, 2H), 6.59 (s, 1H), 4.48-4.43 (q, *J* = 7.2 Hz, 2H), 4.36-4.31 (q, *J* = 7.2 Hz, 2H), 2.50 (s, 3H), 1.92 (s, 3H), 1.46-1.43 (t, *J* = 7.2 Hz, 3H), 1.41-1.38 (t, *J* = 7.2 Hz, 3H); ^{13}C NMR ($CDCl_3$, 100 Hz)

δ 177.6, 165.8, 165.1, 164.0, 157.7, 148.3, 143.0, 139.8, 132.1, 131.6, 130.1, 128.9, 124.4, 120.0, 119.6, 118.1, 59.6, 59.4, 21.9, 20.3, 14.0, 13.9.

***N*,1-Bis(2-chlorophenyl)-1,4-dihydro-2,6-dimethyl-4-oxopyridine-3-carboxamide (2c)** [2]

Colorless crystal (283 mg, 73%); Mp: 182−184 °C (lit. 184−186 °C); ^{1}H NMR ($CDCl_3$, 400 Hz) δ 12.85 (s, 1H), 8.45-8.43 (d, *J* = 8.0 Hz, 1H), 7.64-7.62 (d, *J* = 7.6 Hz, 1H), 7.50-7.51 (m, 2H), 7.39-7.37 (d, *J* = 7.6 Hz, 1H), 7.24-7.20 (m, 2H), 7.01-6.97 (t, *J* = 7.6 Hz, 1H), 6.63 (s, 1H), 2.48 (s, 3H), 1.90 (s, 3H); ^{13}C NMR ($CDCl_3$, 100 Hz) δ 177.6, 164.3, 156.4, 148.3, 137.1, 136.2, 132.7, 131.6, 131.2, 130.9, 129.6, 129.3, 128.8, 127.0, 124.2, 122.8, 119.1, 119.0, 21.0, 19.8.

***N*,1-Bis(3-chlorophenyl)-1,4-dihydro-2,6-dimethyl-4-oxopyridine-3-carboxamide (2d)** [2]

Colorless crystal (298 mg, 77%); Mp: 187−188 °C (lit. 188−189 °C); ^{1}H NMR ($CDCl_3$, 400 Hz) δ 12.88 (s, 1H), 7.88 (s, 1H), 7.57-7.53 (d, *J* = 7.6 Hz, 2H), 7.46-7.42 (t, *J* = 7.6 Hz, 1H), 7.30-7.28 (d, *J* = 8.0 Hz, 1H), 7.21-7.17 (t, *J* = 8.0 Hz, 1H), 7.15-7.13 (d, *J* = 7.6 Hz, 1H), 7.05-7.03 (d, *J* = 7.2 Hz, 1H), 6.58 (s, 1H), 2.46 (s, 3H), 1.91 (s, 3H).; ^{13}C NMR ($CDCl_3$, 100 Hz) δ 177.8, 164.4, 156.2, 148.0, 140.8, 140.0, 136.4, 134.5, 131.3, 130.1, 129.7, 127.8, 126.6, 123.5, 120.1, 119.3, 118.9, 118.5, 21.3, 20.0.

***N*,1-Bis(4-chlorophenyl)-1,4-dihydro-2,6-dimethyl-4-oxopyridine-3-carboxamide (2e)** [1]

Colorless crystal (302 mg, 78%); Mp: 270−272 °C (lit. 269−271 °C); ^{1}H NMR ($CDCl_3$, 400 Hz) δ 12.74 (s, 1H), 7.64-7.62 (d, *J* = 8.8 Hz, 2H), 7.56-7.54 (d, *J* = 8.8 Hz, 2H), 7.26-7.24 (d, *J* = 8.8 Hz, 2H), 7.16-7.13 (d, *J* = 8.8 Hz, 2H), 6.51 (s, 1H), 2.49 (s, 3H), 1.91 (s, 3H); ^{13}C NMR ($CDCl_3$, 100 Hz) δ 177.8, 171.1, 163.9, 156.2, 148.4, 137.9, 137.5, 136.4, 130.8, 129.1, 128.7, 128.5, 121.8, 119.0, 21.8, 20.8.

1,4-Dihydro-*N*,1-bis(2-methoxyphenyl)-2,6-dimethyl-4-oxopyridine-3-carboxamide (2f) [1]

Colorless crystal (321 mg, 85%); Mp: 241−243 °C (lit. 239−241 °C); ^{1}H NMR

($CDCl_3$, 400 Hz) δ 12.56 (s, 1H), 8.51-8.49 (d, *J* = 8.0 Hz, 1H), 7.50-7.48 (d, *J* = 8.0 Hz, 1H), 7.11-7.00 (m, 4H), 6.97-6.90 (m, 2H), 6.50 (s, 1H), 3.94 (s, 3H), 3.80 (s, 3H), 2.44 (s, 3H), 1.86 (s, 3H); ^{13}C NMR ($CDCl_3$, 100 Hz) δ 177.9, 172.5, 164.1, 156.5, 154.3, 149.4, 148.8, 131.6, 128.9, 128.9, 128.1, 123.3, 121.6, 120.9, 120.6, 118.6, 112.3, 110.2, 56.0, 55.8, 20.9, 19.6.

1,4-Dihydro-*N*,1-bis(4-methoxyphenyl)-2,6-dimethyl-4-oxopyridine-3-carboxamide (2g) [2]

Colorless crystal (340 mg, 90%); Mp: 208−210 °C (lit. 211−214 °C); ^{1}H NMR ($CDCl_3$, 400 Hz) δ 12.17 (s, 1H), 7.62-7.60 (d, *J* = 8.8 Hz, 2H), 7.11-7.09 (d, *J* = 8.8 Hz, 2H), 7.03-7.01 (d, *J* = 8.8 Hz, 2H), 6.85-6.83 (d, *J* = 8.8 Hz, 2H), 6.67 (s, 1H), 3.86 (s, 3H), 3.76 (s, 3H), 2.47 (s, 3H), 1.92 (s, 3H); ^{13}C NMR ($CDCl_3$, 100 Hz) δ 177.9, 171.1, 163.8, 16.05, 156.3, 148.8, 132.7, 132.0, 129.0, 122.9, 119.3, 118.4, 115.0, 114.2, 55.6, 55.4, 21.8, 20.9.

***N*,1-Dis(5-chloro-2-methoxyphenyl)-1,4-dihydro-2,6-dimethyl-4-oxopyridine-3-carboxamide (2h)** [1].

Colorless crystal (353 mg, 79%); Mp: 216−218 °C (lit. 215−217 °C); ^{1}H NMR ($CDCl_3$, 400 Hz) δ 12.88 (s, 1H), 8.63-8.61 (d, *J* = 8.4 Hz, 1H), 7.50-7.48 (d, *J* = 7.6 Hz, 1H), 7.20-7.18 (d, *J* = 8.0 Hz, 1H), 7.06-7.04 (d, *J* = 8.8 Hz, 1H), 7.01-6.99 (d, *J* = 8.4 Hz, 1H), 6.83-6.81 (d, *J* = 8.4 Hz, 1H), 6.51 (s, 1H), 3.97 (s, 3H), 3.81 (s, 3H), 2.49 (s, 3H), 1.93 (s, 3H); ^{13}C NMR ($CDCl_3$, 100 Hz) δ 178.0, 164.9, 157.1, 153.0, 149.0, 147.9, 132.2, 130.0, 128.9, 128.7, 127.1, 125.3, 122.1, 119.9, 118.3, 113.2, 111.6, 57.3, 56.9, 20.7, 19.3.

***N*,1-Bis(4-ethoxyphenyl)-1,4-dihydro-2,6-dimethyl-4-oxopyridine-3-carboxamide (2i)** [2]

Colorless crystal (349 mg, 86%); Mp: 220−222 °C (lit. 223−225 °C); ^{1}H NMR ($CDCl_3$, 400 Hz) δ 12.63 (s, 1H), 7.64-7.62 (d, *J* = 8.8 Hz, 2H), 7.10-7.08 (d, *J* = 8.8 Hz, 2H), 7.02-7.69 (d, *J* = 8.8 Hz, 2H), 6-89-6.86 (d, *J* = 8.8 Hz, 2H), 6.51 (s, 1H), 4.11-4.06 (q, *J* = 7.2 Hz, 2H), 4.03-3.98 (q, *J* = 7.2 Hz, 2H), 2.51 (s, 3H), 1.94 (s, 3H), 1.46-1.43 (t, *J* = 7.2 Hz, 3H), 1.40-1.37 (t, *J* = 7.2 Hz, 3H); ^{13}C NMR ($CDCl_3$, 100 Hz)

δ 177.3, 164.5, 159.5, 156.0, 155.5, 149.3, 132.4, 131.8, 129.0, 122.6, 118.7, 118.6, 115.3, 114.0, 64.2, 63.9, 21.9, 20.6, 14.7, 14.6.

1,4-Dihydro-2,6-dimethyl-4-oxo-*N*,1-di-*o*-tolylpyridine-3-carboxamide (2j) [1]
Colorless crystal (249 mg, 72%); Mp: 164−166 °C (lit. 163−165 °C); ^{1}H NMR ($CDCl_3$, 400 Hz) δ 12.48 (s, 1H), 8.14-8.12 (d, J = 8.0 Hz, 1H), 7.45-7.36 (m, 3H), 7.18-7.15 (t, J = 7.6 Hz, 2H), 7.11-7.10 (d, J = 7.6 Hz, 1H), 7.01-6.97 (t, J = 7.6 Hz, 1H), 6.60 (s, 1H), 2.48 (s, 3H), 2.42 (s, 3H), 2.0.2 (s, 3H), 1.85 (s, 3H); ^{13}C NMR ($CDCl_3$, 100 Hz) δ 177.9, 164.1, 156.3, 148.3, 138.6, 137.3, 134.9, 132.0, 130.3, 130.2, 129.0, 128.1, 127.6, 126.2, 123.9, 122.3, 119.1, 119.1, 21.2, 19.9, 18.6, 17.0.

1,4-Dihydro-2,6-dimethyl-4-oxo-*N*,1-di-*m*-tolylpyridine-3-carboxamide (2k) [2]
Colorless crystal (280 mg, 81%); Mp: 155−157 °C (lit. 158−160 °C); ^{1}H NMR ($CDCl_3$, 400 Hz) δ 12.79 (s, 1H), 7.61 (s, 1H), 7.53-7.46 (m, 2H), 7.35-7.33 (d, J = 7.6 Hz, 1H), 7.22-7.18 (t, J = 7.6 Hz, 1H), 7.02-7.00 (d, J = 7.6 Hz, 2H), 6.90-6.88 (d, J = 7.6 Hz, 1H), 6.52 (s, 1H), 2.49 (s, 3H), 2.45 (s, 3H), 2.36 (s, 3H), 1.97 (s, 3H); ^{13}C NMR ($CDCl_3$, 100 Hz) δ 177.9, 164.3, 156.8, 149.1, 141.1, 139.5, 139.0, 138.5, 130.0, 129.9, 128.4, 128.1, 125.1, 124.7, 120.7, 118.8, 118.7, 118.1, 21.8, 21.6, 20.9, 19.9.

1,4-Dihydro-2,6-dimethyl-4-oxo-*N*,1-di-*p*-tolylpyridine-3-carboxamide (2l) [2]
Colorless crystal (287 mg, 83%); Mp: 241−242 °C (lit. 241−243 °C); ^{1}H NMR ($CDCl_3$, 400 Hz) δ 12.25 (s, 1H), 7.61-7.59 (d, J = 8.0 Hz, 2H), 7.35-7.33 (d, J = 7.6 Hz, 2H), 7.11-7.07 (t, J = 8.0 Hz, 4H), 6.70 (s, 1H), 2.46 (s, 3H), 2.43 (s, 3H), 2.28 (s, 3H), 1.90 (s, 3H); ^{13}C NMR ($CDCl_3$, 100 Hz) δ 177.8, 164.5, 156.2, 148.7, 140.4, 136.8, 136.3, 133.3, 131.0, 129.3, 127.2, 120.6, 118.9, 118.7, 21.7, 21.2, 20.8, 20.7.

1,4-Dihydro-2,6-dimethyl-*N*,1-bis(2,4-dimethylphenyl)-4-oxopyridine-3-carboxamide (2m) [2].
Colorless crystal (292 mg, 78%); Mp: 161−163 °C (lit. 158−160 °C); ^{1}H NMR ($CDCl_3$, 400 Hz) δ 12.41 (s, 1H), 8.00-8.98 (d, J = 8.0 Hz, 1H), 7.17-7.14 (d, J = 11.2 Hz, 2H), 6.98-6.95 (t, J = 7.6 Hz, 3H), 6.53 (s, 1H), 2.48 (s, 3H), 2.38 (s, 6H), 2.26 (s, 3H), 1.97 (s, 3H), 1.85 (s, 3H), 1.83 (s, 3H); ^{13}C NMR ($CDCl_3$, 100 Hz) δ 178.0,

164.2, 156.4, 148.3, 140.4, 136.1, 134.8, 134.5, 133.3, 132.6, 130.9, 128.9, 128.7, 127.3, 126.7, 122.3, 119.0, 21.2, 21.1, 20.8, 19.9, 18.5, 16.9.

***N*,1-Dibenzyl-1,6-dihydro-2,4-dimethyl-6-oxopyridine-3-carboxamide (2n)** [1].

Colorless crystal (142 mg, 82%); Mp: 94-96 °C (lit. 96−98 °C); ^{1}H NMR ($CDCl_3$, 400 Hz) δ 7.31-7.20 (m, 8H), 7.03-7.02 (d, *J* = 5.6 Hz, 2H), 7.02 (s, 1H), 6.23 (s, 1H), 5.13 (s, 2H), 4.52-4.51 (d, *J* = 5.6 Hz, 2H), 2.18 (s, 3H), 2.12 (s, 3H); ^{13}C NMR ($CDCl_3$, 100 Hz) δ 167.1, 162.8, 148.2, 143.3, 137.8, 135.6, 128.8, 128.7, 128.1, 127.7, 127.5, 126.4, 119.3, 116.9, 47.1, 44.0, 19.6, 17.7.

***N*,1-Bis(4-chlorobenzyl)-1,6-dihydro-2,4-dimethyl-6-oxopyridine-3-carboxamide (2o)** [1].

Colorless crystal (329 mg, 81%); Mp: 150−152 °C (lit. 151−153 °C); ^{1}H NMR ($CDCl_3$, 400 Hz) δ 7.36-7.34 (d, *J* = 8.0 Hz, 2H), 7.11 (s, 1H), 7.02-7.00 (d, *J* = 8.0 Hz, 2H), 6.82−6.77 (m, 4H), 6.22 (s, 1H), 5.10 (s, 2H), 4.50-4.49 (d, *J* = 5.6 Hz, 2H), 3.75 (s, 3H), 3.74 (s, 3H), 2.19 (s, 3H), 2.12 (s, 3H); ^{13}C NMR ($CDCl_3$, 100 Hz) δ 167.1, 162.9, 158.0, 148.2, 143.1, 130.6, 130.1, 128.7, 127.2, 127.0, 119.8, 117.4, 114.5, 114.0, 55.6, 55.5, 44.8, 43.9, 19.7, 17.7.

References

1. Pierce, J. B.; Ariyan, Z. S.; Ovenden, G. S. *J. Med. Chem.* **1982**, *25*, 131–136.

2. Zhang, Z. G.; Fang, S. L.; Liu,Q. F.; Zhang, G. S. *J. Org. Chem.* **2012**, *77*, 7665–7670.
